# Supplementary material for: Splice-Junction-Based Mapping of Alternative Isoforms in the Human Proteome
Source: Cell Rep. Author manuscript; Available in PMC 2020 Jan 15. (PMC6961840; doi:10.1016/j.celrep.2019.11.026)
Supplement: 2 [file NIHMS1546469-supplement-2.zip › DF1/PXD010154/AdrenalGland/AdrenalGland_11_RBBP4_GPDAVEERVINEEYK.pdf]

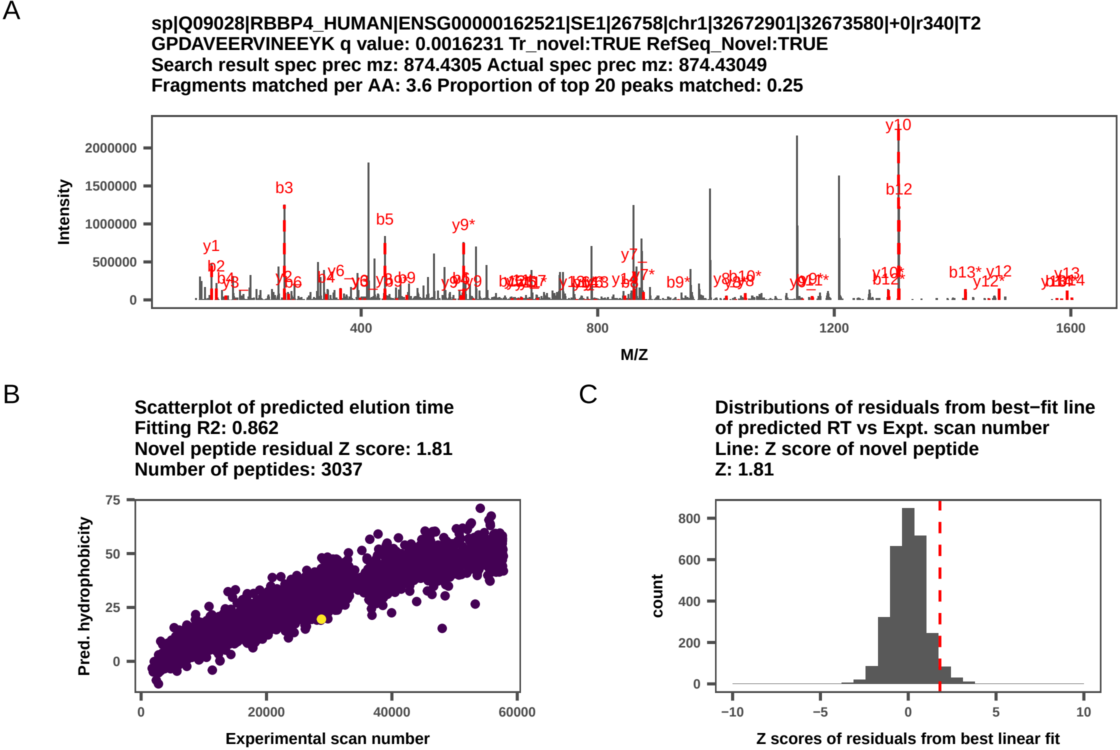
A
sp|Q09028|RBBP4_HUMAN|ENSG00000162521|SE1|26758|chr1|32672901|32673580|+0|r340|T2
GPDAVEERVINEEYK q value: 0.0016231 Tr_novel:TRUE RefSeq_Novel:TRUE
Search result spec prec mz: 874.4305 Actual spec prec mz: 874.43049
Fragments matched per AA: 3.6 Proportion of top 20 peaks matched: 0.25
Intensity
2000000
1500000
1000000
500000
0
y10
b12
b3
b5
y9*
y1
b2
b13* y12
y12*
M/Z
400
800
1200
1600
B
Scatterplot of predicted elution time
Fitting R2: 0.862
Novel peptide residual Z score: 1.81
Number of peptides: 3037
Pred. hydrophobicity
75
50
25
0
0
20000
40000
60000
Experimental scan number
C
Distributions of residuals from best−fit line
of predicted RT vs Expt. scan number
Line: Z score of novel peptide
Z: 1.81
count
800
600
400
200
0
−10
−5
0
5
10
Z scores of residuals from best linear fit
